# Lymph node status and its impact on the prognosis of left‐sided and right‐sided colon cancer: A SEER population‐based study

**DOI:** 10.1002/cam4.4357

**Published:** 2021-10-26

**Authors:** Yadi Huang, Linlin Ji, Jialong Zhu, Xiaobei Mao, Siqi Sheng, Shuai Hao, Dan Xiang, Jiani Guo, Gongbo Fu, Mengxi Huang, Zengjie Lei, Xiaoyuan Chu

**Affiliations:** ^1^ Department of Medical Oncology Jinling Hospital The First School of Clinical Medicine Southern Medical University Nanjing China; ^2^ Department of Medical Oncology Jinling Hospital Medical School of Nanjing University Nanjing University Nanjing China; ^3^ Department of General Surgery Jinling Hospital Medical School of Nanjing University Nanjing China; ^4^ Department of Medical Oncology Jinling Hospital Nanjing Medical university Nanjing China

**Keywords:** left‐sided colon cancer, lymph node status, nomogram, prognosis, right‐sided colon cancer

## Abstract

**Background:**

Some significant differences exist between the outcomes of left‐ and right‐sided colon cancer patients. The presence of nodal metastases is a critical prognostic factor, especially in the absence of distant metastasis. Our research studied the lymph nodes status of left‐ and right‐sided colon cancer patients to determine the influence of this factor on prognosis.

**Methods:**

Our data were obtained from the Surveillance, Epidemiology and End Results (SEER) database. We used the chi‐square test to analyze the clinicopathological characteristics. The X‐tile program was adopted to acquire optimal cutoff points of lymph node index. Kaplan–Meier curves were used to analyze prognosis and multivariate Cox regression models were performed to identify the independent factors associated with survival. Nomograms were built to predict the overall survival of patients, Harrell's C‐index and calibration plots were used to validate the nomograms.

**Results:**

The study included 189,941 patients with colon cancer without metastasis (left 69,885, right 120,056) between 2004 and 2015. There are more patients with adequate examined lymph nodes in right‐sided. Lymph node status in patients with right colon cancer has a more significant impact on the risk of death. LODDS (C‐index: 0.583; AIC: 6875.4) was used to assess lymph node status. The nomograms showed that lymph node status was the main factor to predict the outcome in right‐sided colon patients.

**Conclusions:**

The influence of lymph node status on predicting prognosis is significantly different between patients with left and right colon cancer without metastasis. The tumor site needs to be considered when lymph node status is used to assess the outcome of patients.

## Graphical abstract

The effect of lymph node status on predicting prognosis differed significantly between left‐sided and right‐sided colon cancer patients. The tumor site needs to be considered when lymph node status is used to assess the outcome of patients.

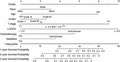

## 1 INTRODUCTION

The mortality rate of colon cancer has been increasing over years, and the overall prognosis of patients with colon cancer remains poor.^1^ Increasing evidence suggests that different sites of colon cancers are distinct in the aspect of molecular pathogenesis, histology, response to treatment, and prognosis.^2^ There are many factors which could influence the prognosis of colon cancer, including age, histological type, metastatic lymph node status, and treatment.^3^ For those cancer patients without distant metastasis, the presence of nodal metastases represents an important determinant of prognosis.^4^ Studies have shown that lymph node status has different effects on left‐ and right‐sided colon cancer patients. Yang^5^ suggested that the 12‐node standard of the National Comprehensive Cancer Network (NCCN) guidelines for the examination of lymph nodes, version 2.2021, for colon cancer^6^ is not equally applicable to all parts of colon cancer. Moreover, right‐sided colon cancer is less responsive to immunotherapy, including small‐molecule inhibitors targeting the immune escape.^7^ Previous studies also suggest that immune responses of metastatic lymph nodes are restricted, which may lead to evasion of immune surveillance.^8^ These results suggest that lymph node status differs in left‐ and right‐sided colon cancer patients. The differences in lymph node status and its impact on outcome between different sites of colon cancer patients have not been examined in detail.

The AJCC seventh edition recommends that lymph node status should be assessed by N staging system, patients were divided into N0, N1, N2 three stages according to the number of metastatic lymph nodes. However, on account of various factors such as tumor site, examination of lymph node status is not adequate in some patients,^9^ which could disturb lymph node staging to predict patient prognosis. Michal found that examined lymph nodes was less in left‐sided colon cancer patients, which might result in incorrect lymph node staging and thus inaccurate prediction of long‐term outcomes.^10^ Two alternative lymph node staging systems are frequently used: lymph node rate (LNR) is the ratio of the metastatic lymph nodes to the examined lymph nodes; however, it cannot differentiate patients without nodal metastases, and it cannot stratify patients whose examined nodes all are positive (LNR = 1). The log odds of metastatic lymph nodes (LODDS) are the log of ratio between the metastatic lymph nodes to the negative lymph nodes. The role of LODDS has been investigated in different types of cancer, and most studies have confirmed its relevance to predicting the outcome of patients.^11^, ^12^, ^13^ Our research aimed to identify the most accurate lymph node staging system to assess lymph node status, and then systematically analyze the influence of lymph node status on prognosis between left‐ and right‐sided colon cancer without metastasis.

## 2 METHODS

### 2.1 Data source and exclusion criteria

**FIGURE 1:**
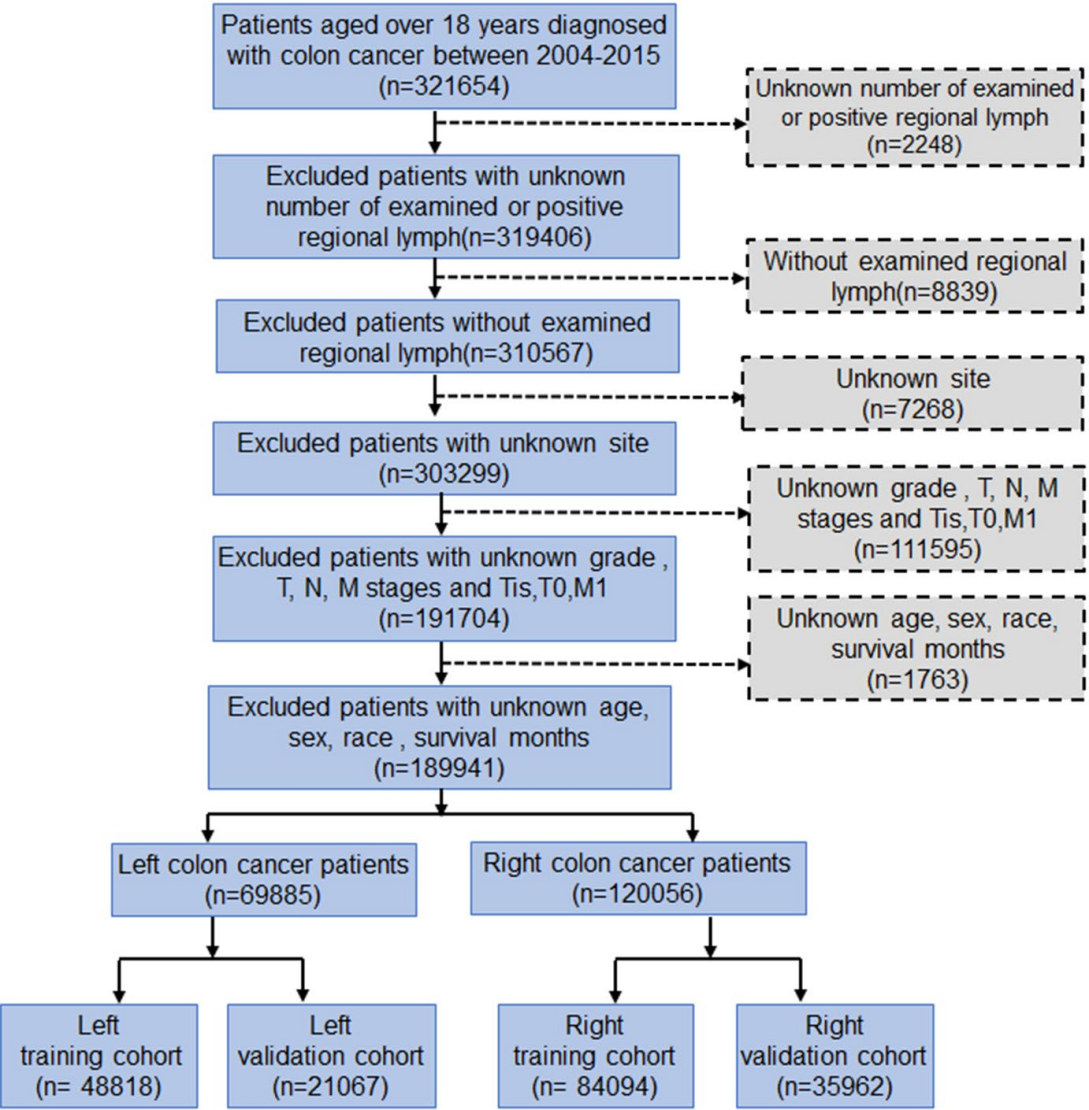
Flow diagram of eligible patients selected from the SEER Database

Our data were obtained from the Surveillance, Epidemiology, and End Results (SEER) cancer registry. On account of some data on lymph node were not available until 2004 and some patients with distant metastasis might receive palliative resection thus not get lymph node excision. We chose patients >18 years old diagnosed with colon cancer without metastasis between 2004 and 2015. Patients who met the following criteria were excluded: (a) unknown number of examined or positive regional lymph nodes; (b) without examined regional lymph nodes; (c) unknown or non‐specific tumor primary site; (d) unknown grade and T, N, and M stages; (e) incomplete demographic information on age, sex, race and survival months. Finally, 189,941 patients were chosen in our study. Patients were categorized into two groups: those diagnosed with left‐sided colon cancer (LCC; splenic flexure, descending colon, and sigmoid colon) and those diagnosed with right‐sided colon cancer (RCC; cecum, ascending colon, hepatic flexure, and transverse colon), which included 69,885 and 120,056 patients, respectively. Figure 1 depicts the selection process.

### 2.2 Statistical analysis

LNR is the ratio of metastatic lymph nodes to the examined lymph nodes. The LODDS was calculated by the computational formula: Ln[(MLN+0.5)/(TNLNMLN+0.5)],^14^ MLN presents the number of metastatic lymph nodes, and TNLN is the abbreviation of total number of examined lymph nodes. We adopted the X‐tile program to get the optimal LNR and LODDS cutoff points, and used chi‐square test to compare the clinicopathological characteristics of left‐ and right‐side colon cancer. Kaplan–Meier curves were used to analyze prognosis. We chose the Harrell's concordance index (C‐index) and Akaike's Information Criterion (AIC) to evaluate the accuracy of different lymph node indexes. Lower AIC represents a better fit and a higher C‐index means a better discrimination ability.^15^ Patients were divided into a training set and a validation set (Figure 1). We adopted multivariate Cox regression models to analyze independent prognostic factors in the training set. The independent factors were included to construct nomograms to predict overall survival (OS). We assessed the nomogram in both training set and validation set. We chose C‐index and the calibration plot to evaluate the nomogram. When C‐index reaches 0.50, representing the nomogram has discriminative ability^16^; in calibration plot, when the results fall at diagonal line, it shows a perfectly calibrated model.^17^ Our analyses were performed by R statistical software (version 4.0.3).

## 3 RESULTS

### 3.1 Baseline characteristics of left‐sided and right‐sided colon cancer patients

Of 189,941 eligible patients included in the study, 69,885 (36.8%) were LCC and 120,056 (63.2%) were RCC. Firstly, we compared the clinicopathological characteristics of the LCC and RCC patients (Table 1). Patient age, sex, tumor grade, TNLN, and LODDS were different in LCC and RCC. The proportion of patients >65 years old was higher in the RCC group than LCC group (69.72% vs. 52.53%, 0.001). There were more women than men in the RCC group (54.23% vs. 45.77%, 0.001), and the opposite pattern was observed in the LCC group (46.53% vs. 53.47%, 0.001). Most patients had moderately differentiated tumors (LCC 76.63% and RCC 68.11%, 0.001). The degree of differentiation was poorer in the RCC group, which had more patients with poorly differentiated (LCC 12.2% and RCC 19.98%, 0.001) or undifferentiated tumors (LCC 1.49% and RCC 3.06%, 0.001). NCCN Guidelines Version 2.2021 for colon cancer recommends that colon cancer patients should be examined ≥12 lymph nodes to guarantee accurate staging.^6^ Even so, adequate examination of lymph nodes did not occur in some patients, especially in the LCC group (LCC 30.5% and RCC 18.71%, 0.001).

**TABLE 1:** Baseline characteristics for left‐sided and right‐sided colon cancer patients

Variables	Left‐sided (*n* = 69,885)	Right‐sided (*n* = 120,056)	*p*
	*n* (%)	*n* (%)	
Age			
<65	33171 (47.47)	36349 (30.28)	<0.001
≥65	36714 (52.53)	83707 (69.72)	
Sex			
Male	37368 (53.47)	54946 (45.77)	<0.001
Female	32517 (46.53)	65110 (54.23)	
Race			
White	54546 (78.05)	98860 (82.34)	<0.001
Black	7836 (11.21)	13733 (11.44)	
Other (American Indian/AK Native, Asian/Pacific Islander)	7503 (10.74)	7463 (6.22)	
Grade			
I (Well)	6772 (9.69)	10624 (8.85)	<0.001
II (Moderately)	53550 (76.63)	81766 (68.11)	
III (Poorly)	8525 (12.2)	23992 (19.98)	
IV (Undifferentiated)	1038 (1.49)	3674 (3.06)	
T			
T1	11550 (16.53)	14381 (11.98)	<0.001
T2	11060 (15.83)	21244 (17.7)	
T3	38676 (55.34)	69357 (57.77)	
T4	8599 (12.3)	15074 (12.56)	
N			
N0	43414 (62.12)	77767 (64.78)	<0.001
N1	18085 (25.88)	27308 (22.75)	
N2	8386 (12)	14981 (12.48)	
TNLN			
<12	21313 (30.5)	22468 (18.71)	<0.001
≥12	48572 (69.5)	97588 (81.29)	
LNR			
LNR < 0.1	52241 (74.75)	93435 (77.83)	<0.001
0.1 ≤ LNR < 0.3	10505 (15.03)	15816 (13.17)	
LNR ≥ 0.3	7139 (10.22)	10805 (9)	
LODDS			
LODDS < −3.2	29544 (42.28)	63438 (52.84)	<0.001
−3.2 ≤ LODDS < −0.9	32324 (46.25)	44697 (37.23)	
LODDS ≥ −0.9	8017 (11.47)	11921 (9.93)	

### 3.2 The impact of lymph node status to predict prognosis is more significant in right‐sided colon cancer patients

**FIGURE 2:**
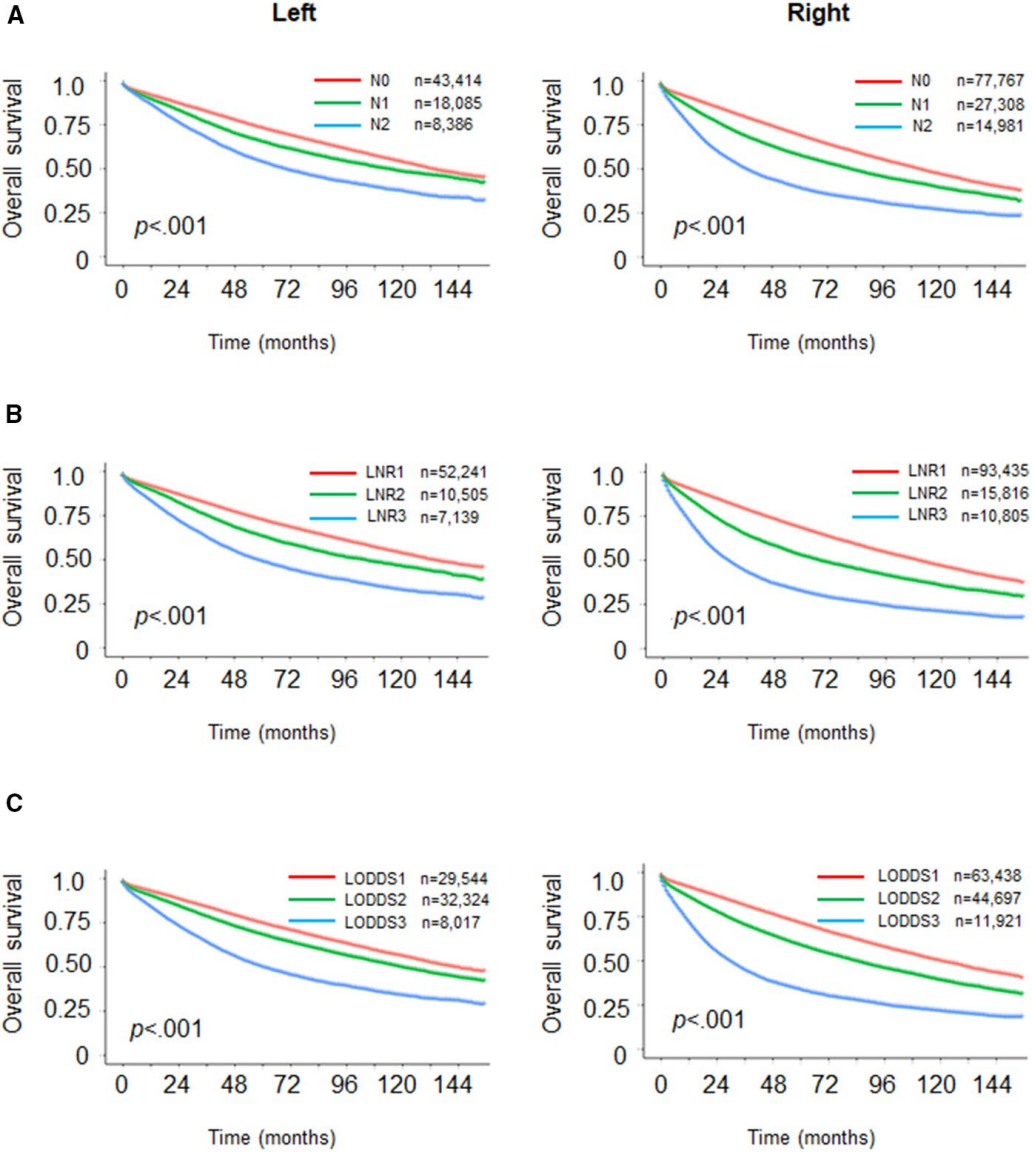
Kaplan–Meier curves of the impact of N stage (A), LNR(B), LODDS (C) in LCC and RCC cohorts on OS

Metastatic lymph node is a risk prognostic factor for colon cancer. Currently, the AJCC N classification, which depends on the absolute number of metastatic lymph nodes, is the most universally clinical staging system.^18^ However, we found that some patients did not have an adequate examination of lymph nodes (Table 1), which might influence the accuracy of N staging to predict the outcome of patients. Therefore, it is necessary to identify a better lymph node staging system. We analyzed survival in relation to three lymph node staging systems. To group patients according to lymph node status, we used the X‐tile program to obtain optimum cutoff values (LNR1: LNR < 0.1, LNR2: 0.1 ≤ LNR < 0.3, LNR3: LNR ≥ 0.3; LODDS1: LODDS < −3.2, LODDS2: −3.2 ≤ LODDS < −0.9, LODDS3: LODDS ≥ −0.9) (Figure S1). The patients with LODDS < −3.2 was more in RCC than in LCC (52.84% vs. 42.28%, *p* < 0.001), and there were more patients with LODDS ≥ −0.9 in the LCC group (11.47% vs. 9.93%, *p* < 0.001) (Table 1). In Figure 2, patients in the higher N stage group, LNR group, or LODDS group had poorer OS regardless of LCC or RCC. There were significant differences between LCC and RCC, namely, the differences among three lymph node staging subs were greater in RCC than in LCC (Figure 2), and no matter in which staging system, the outcome of LCC patients was better. In addition, the LCC and RCC groups showed different 5‐year OS rates on N stage subs (LCC: N0 72.87%, N1 65.04%, N2 53.52%; RCC: N0 68.89%, N1 57.42%, N2 38.97%); and similar results were obtained in LNR and LODDS subs [LNR (LCC: LNR1 72.41%, LNR2 63.12%, LNR3 48.84%; RCC: LNR1 67.87%, LNR2 52.39%, LNR3 32.19%); LODDS (LCC: LODDS1 74.54%, LODDS2 68.04%, LODDS3 49.8%; RCC: LODDS1 71.11%, LODDS2 58.6%, LODDS3 33.45%)] (Figure 2).

We separated patients in two cohorts according to TNLN < 12 and TNLN ≥ 12 for further analysis. Based on the LODDS cutoff values calculated from the entire cohort, when LODDS was <−3.2, there were no patients in the TNLN < 12 group. This is probably due to its calculation formula, in TNLN < 12 group, the minimum value of LODDS is bigger than −3.2. To prevent bias in subsequent statistical analyses, we calculated the LODDS cutoff values in the TNLN < 12 and TNLN ≥ 12 groups (Figure S2) and divided them into three subs (LODDS’1, LODDS’2, LODDS’3; LODDS’’1, LODDS’’2, LODDS’’3). No matter in TNLN < 12 or TNLN ≥ 12 cohorts, the results were similar to the overall cohort. In addition, patients with TNLN < 12 had a poorer outcome regardless of LCC or RCC, which suggested that TNLN < 12 is a predicting factor of poor prognosis (Figures S3 and S4).

### 3.3 LODDS shows the best prognostic accuracy among staging systems

For every staging system, the accuracy to predict the prognosis of patients is of utmost significance.^12^ We chose C‐index and AIC to evaluate the accuracy of three different lymph node staging systems (Table 2). In the entire cohort of patients, LODDS (C‐index: 0.583; AIC: 6875.4) performed better than other staging systems. According to the result of additional analyses in different TNLN cohorts and different sites (LCC and RCC), we found LODDS maintained the best performance in TNLN < 12 group, while in TNLN ≥ 12 group, the C‐index of LODDS and N stage showed no difference.

**TABLE 2:** Prognostic accuracy of different lymph node staging systems

	ALL		Left		Right	
	C‐index	AIC	C‐index	AIC	C‐index	AIC
ALL						
N (categorical)	0.572	1799612	0.554	548164.6	0.581	1151195
LNR (categorical)	0.57	1798309	0.557	547730.1	0.578	1150064
LODDS (categorical)	0.583	1797885	0.566	547674.7	0.597	1149417
N (continuous)	0.573	1799531	0.555	548083.5	0.583	1151314
LNR (continuous)	0.577	1797270	0.56	547494.8	0.588	1149180
LODDS (continuous)	0.599	1796015	0.578	547246.2	0.615	1147827
TNLN < 12						
N (categorical)	0.56	470603.6	0.553	188036.7	0.567	250120.6
LNR (categorical)	0.559	470598.3	0.554	187998.5	0.565	250135.7
LODDS’ (categorical)	0.563	470547.3	0.558	187985	0.572	250044
N (continuous)	0.56	470483.8	0.554	188019.3	0.567	250044.2
LNR (continuous)	0.562	470366.1	0.556	187947.4	0.569	249943.1
LODDS (continuous)	0.571	470325	0.567	187893.4	0.582	249899
TNLN ≥ 12						
N (categorical)	0.58	1232970	0.56	325322.3	0.59	840715.3
LNR (categorical)	0.572	1232321	0.556	325158	0.58	840169.5
LODDS’’ (categorical)	0.58	1232269	0.56	325183.2	0.59	840036.8
N (continuous)	0.583	1232619	0.563	325146.6	0.593	840623.7
LNR (continuous)	0.585	1231285	0.564	324920.1	0.595	839348.3
LODDS (continuous)	0.598	1230932	0.573	324951	0.612	838826.2

To avoid the impact of different categorical cutoff values, we further repeated our studies via the continuous variables. We used the account of positive lymph nodes to define the continuous N stage. Our results indicated that the LODDS was better than other staging systems both in different sites and different TNLN (Table 2).

### 3.4 Lymph node status accounts for the most powerful predictor to prognosis in RCC according to the nomogram

**FIGURE 3:**
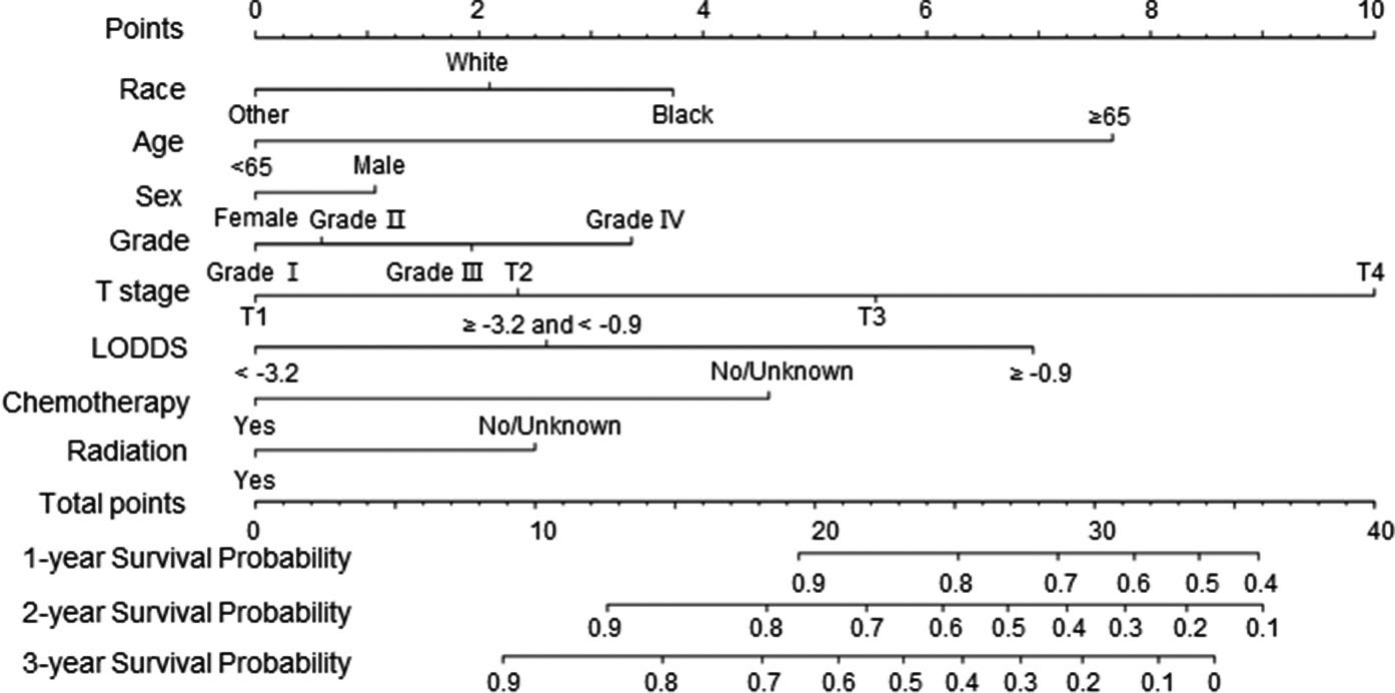
Prediction of 1‐, 3‐, and 5‐year OS of LCC patients via nomograms

It is reported that nomograms can predict survival of patients more accurately than TNM staging in most cancer types.^19^ In this study, LODDS was more accurate than other stage systems (Table 2). Therefore, in the Cox proportional hazard model, we used LODDS to evaluate lymph node metastasis status. In Table 3, the univariate Cox model indicated race, age, sex, grade, T stage, LODDS, chemotherapy, and radiation were factors associated with OS in the LCC training set. These factors were then considered in the multivariate analysis, which showed they were all independent predictors (Table 3). We used these factors to construct the nomogram to predict OS of LCC patients (Figure 3).

**TABLE 3:** Univariable and multivariable Cox proportional hazard analysis of overall survival for left‐sided colon cancer patients

Variables	Univariate analysis of OS			Multivariate analysis of OS		
	Hazard ratio	95% CI	*p*	Hazard ratio	95% CI	*p*
Age						
<65	Ref			Ref		
≥65	2.95	2.86–3.05	<0.001	2.74	2.65–2.83	<0.001
Sex						
Male	Ref			Ref		
Female	0.9	0.88–0.93	<0.001	0.87	0.84–0.89	<0.001
Race						
White	Ref			Ref		
Black	1.11	1.06–1.16	<0.001	1.25	1.19–1.31	<0.001
Other (American Indian/AK Native, Asian/Pacific Islander)	0.73	0.7–0.77	<0.001	0.76	0.72–0.8	<0.001
Grade						
I (Well)	Ref			Ref		
II (Moderately)	1.21	1.15–1.28	<0.001	1.08	1.02–1.14	0.005
III (Poorly)	1.66	1.55–1.76	<0.001	1.29	1.19–1.35	<0.001
IV (Undifferentiated)	1.99	1.76–2.24	<0.001	1.56	1.38–1.75	<0.001
T						
T1	Ref			Ref‐		
T2	1.41	1.33–1.5	<0.001	1.36	1.28–1.45	<0.001
T3	2.06	1.96–2.16	<0.001	2.07	1.97–2.18	<0.001
T4	3.51	3.31–3.72	<0.001	3.73	3.51–3.96	<0.001
LODDS						
LODDS < −3.2	Ref			Ref		
−3.2 ≤ LODDS < −0.9	1.25	1.21–1.29	<0.001	1.41	1.36–1.46	<0.001
LODDS ≥ −0.9	2.12	2.03–2.21	<0.001	2.5	2.38–2.61	<0.001
Chemotherapy						
No/Unknown	Ref			Ref		
Yes	0.71	0.69–0.73	<0.001	0.55	0.53–0.57	<0.001
Radiation						
No/Unknown	Ref			Ref		
Yes	1.23	1.12–1.34	0.021	1.39	1.27–1.52	<0.001
Surgery						
No/Unknown	Ref					
Yes	0.8	0.4–1.59	0.519			

**FIGURE 4:**
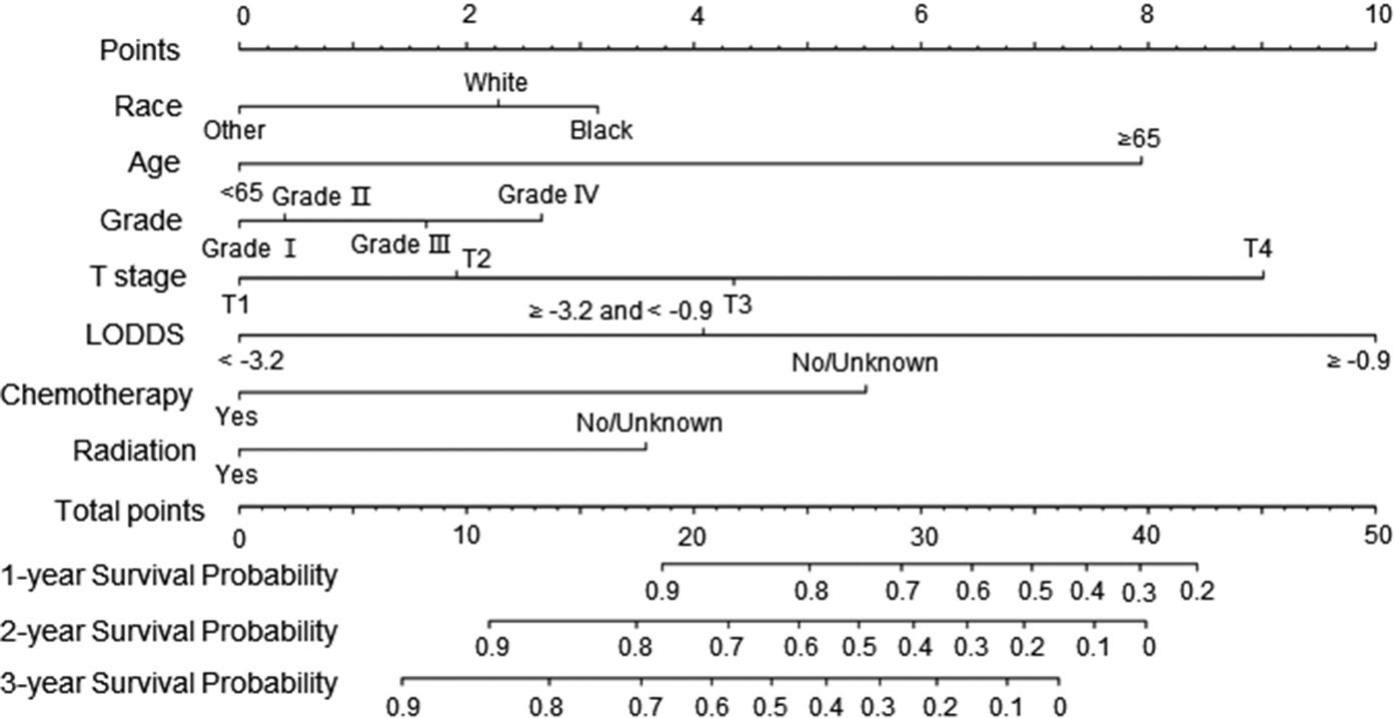
Prediction of 1‐, 3‐, and 5‐year OS of RCC patients via nomograms

In Table 4, the univariate and multivariate Cox model indicated race, age, grade, T stage, LODDS, chemotherapy, and radiation were independent elements in the RCC training set, and we used them to construct the nomogram to predict OS of RCC patients (Figure 4). Compared with the nomogram in LCC, in which T stage was the leading risk factor, LODDS was the leading factor in RCC.

**TABLE 4:** Univariable and multivariable Cox proportional hazard analysis of overall survival for right‐sided colon cancer patients

Variables	Univariate analysis of OS			Multivariate analysis of OS		
	Hazard ratio	95% CI	*p*	Hazard ratio	95% CI	*p*
Age						
<65	Ref			Ref		
≥65	2.72	2.64–2.79	<0.001	2.51	2.44–2.58	<0.001
Sex						
Male	Ref					
Female	0.98	0.96–1	0.05			
Race						
White	Ref			Ref		
Black	0.91	0.88–0.94	<0.001	1.11	1.07–1.14	<0.001
Other (American Indian/AK Native, Asian/Pacific Islander)	0.74	0.71–0.78	<0.001	0.77	0.73–0.81	<0.001
Grade						
I (Well)	Ref			Ref		
II (Moderately)	1.16	1.11–1.2	<0.001	1.05	1.01–1.09	0.021
III (Poorly)	1.61	1.55–1.68	<0.001	1.21	1.16–1.26	<0.001
IV (Undifferentiated)	1.82	1.71–1.95	<0.001	1.36	1.27–1.46	<0.001
T						
T1	Ref			Ref		
T2	1.29	1.23–1.35	<0.001	1.25	1.19–1.31	<0.001
T3	1.71	1.64–1.77	<0.001	1.66	1.59–1.72	<0.001
T4	3.03	2.9–3.16	<0.001	2.84	2.71–2.97	<0.001
LODDS						
LODDS < −3.2	Ref			Ref		
−3.2 ≤ LODDS < −0.9	1.46	1.43–1.49	<0.001	1.6	1.57–1.64	<0.001
LODDS ≥ −0.9	2.86	2.78–2.95	<0.001	3.18	3.08–3.29	<0.001
Chemotherapy						
No/Unknown	Ref			Ref		
Yes	0.73	0.72–0.75	<0.001	0.53	0.51–0.54	<0.001
Radiation						
No/Unknown	Ref			Ref		
Yes	1.39	1.25–1.54	<0.001	1.51	1.36–1.69	<0.001
Surgery						
No/Unknown	Ref					
Yes	0.7	0.47–1.04	0.074			

Further studies were performed to establish the nomograms of TNLN < 12 and TNLN ≥ 12 in different sites of colon cancer (Figures S5 and S6). Compared with the TNLN < 12 group, LODDS had the greatest effect among the prognostic factors in the TNLN ≥ 12 group regardless of LCC or RCC (Figure S6). The results suggested that in colon cancer patients who undergo adequate examination of lymph nodes, lymph node status accounts for the most critical elements to predict patient outcome.

### 3.5 Validation in the nomograms of LCC and RCC

**FIGURE 5:**
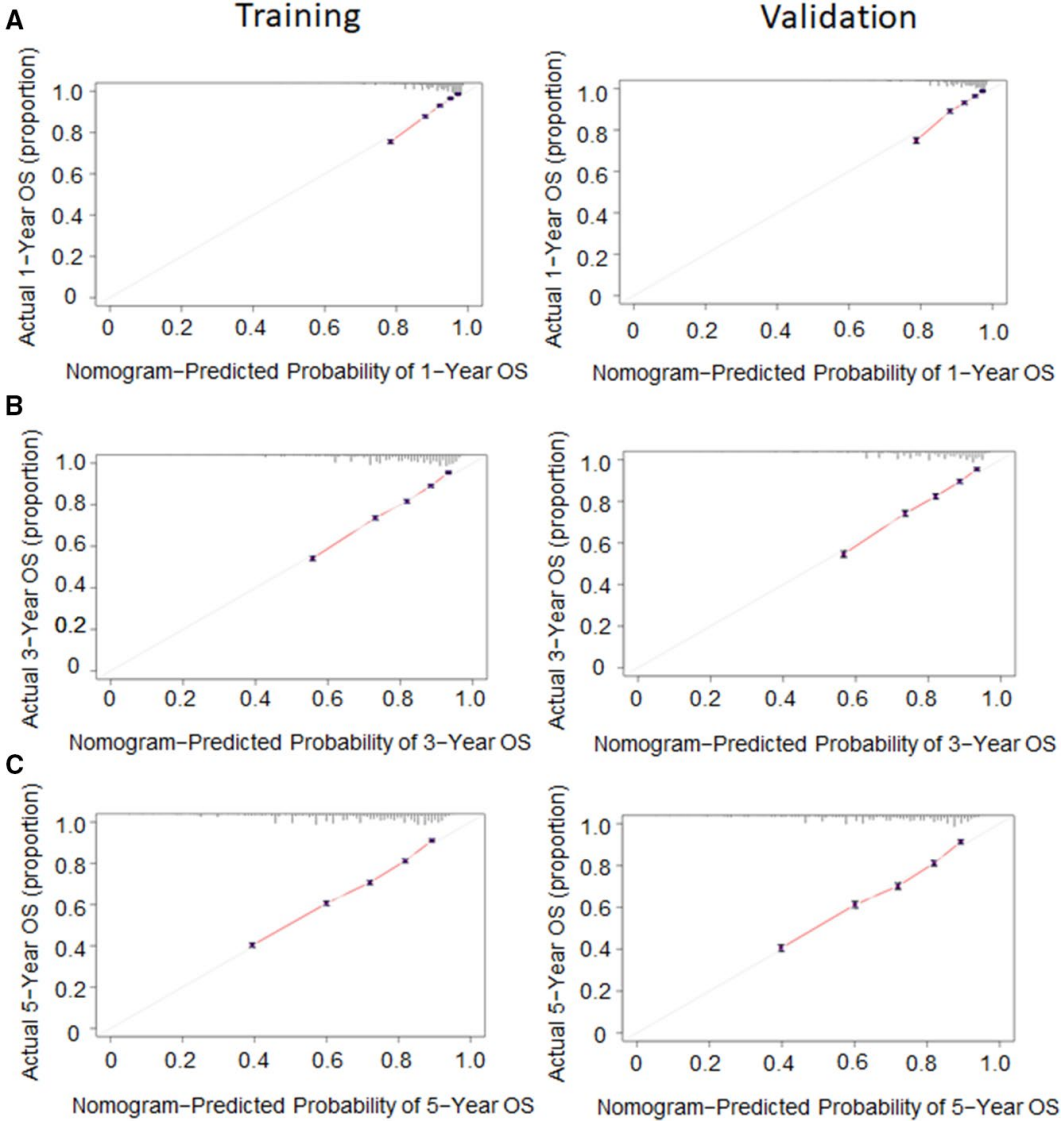
Calibration plots of predicted and actual 1‐(A), 3‐(B), and 5‐year OS(C) predictions for LCC patients in training set and validation set

**FIGURE 6:**
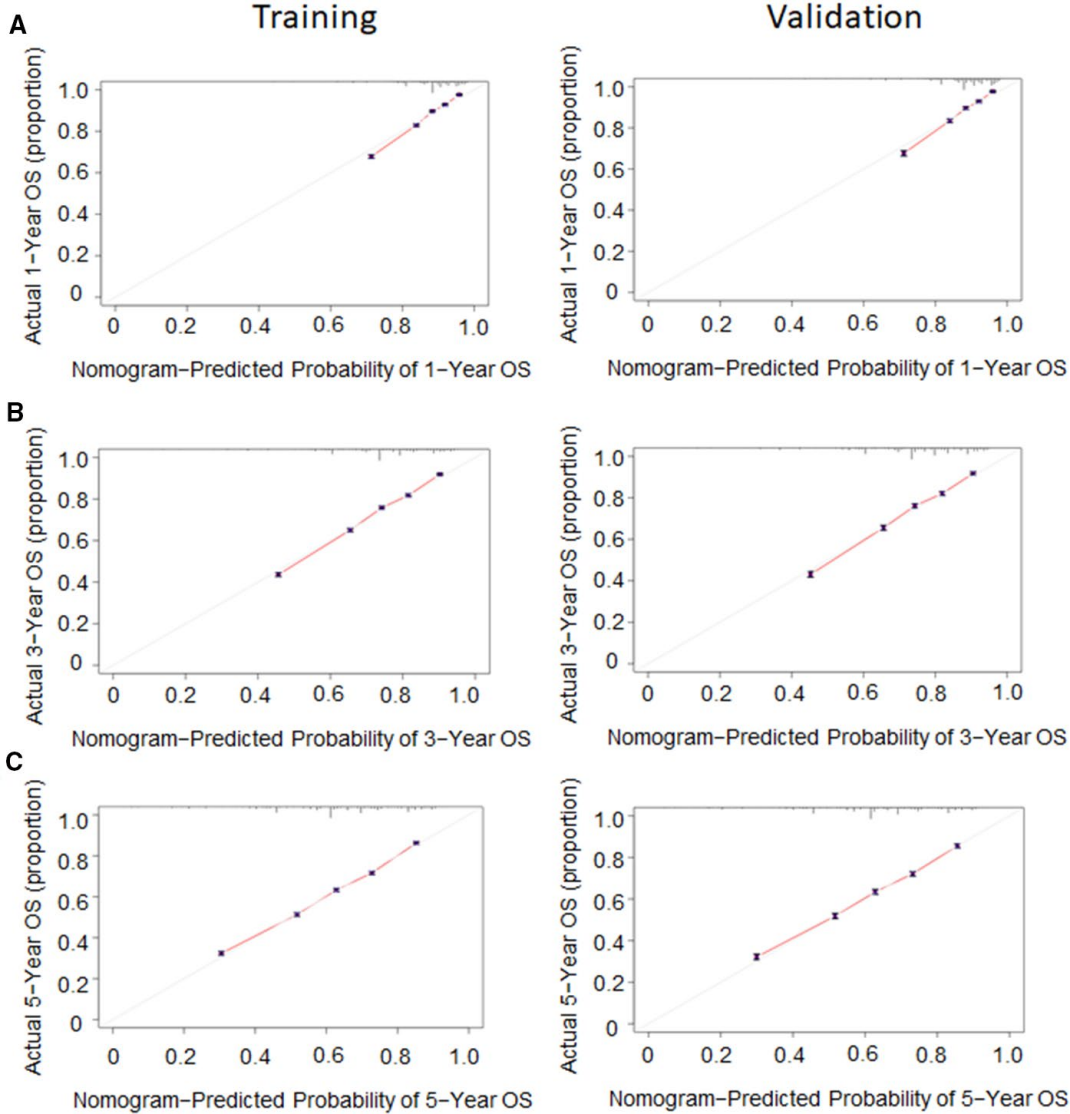
Calibration plots of predicted and actual 1‐(A), 3‐(B), and 5‐year OS(C) predictions for RCC patients in training set and validation set

We adopted a training set to internally validate the nomogram we constructed. The Harrell's C‐index can indicate the discriminatory ability of the nomogram. In LCC, the C‐index was 0.712 (95% CI: 0.709–0.716) in the training set. Similarly, the C‐index was 0.713 (95% CI: 0.707–0.719) in the external validation set. The C‐index of RCC was 0.689 (95% CI: 0.686–0.692) and 0.688 (95% CI: 0.683–0.692) in the training and external validation sets, respectively. Our results indicated the nomograms could accurately predict OS in LCC and RCC. In addition, we also used the calibration plots to assess the nomogram internally and externally, the results indicated that the nomograms predicted an OS rate that closely corresponded to the actual survival rate. (Figures 5 and 6).

## 4 DISCUSSION

Over the past few years, abounding researchers have studied the clinicopathological characteristics of colon cancer in different sites.^20^ LCC and RCC may be two different entities, as they show epidemiological, clinical, and molecular biological differences.^2^ Some authors assumed that this may be a result of their distinct embryological origin.^21^ Nevertheless, these differences might influence the outcome of patients. In this study, we analyzed the characteristics of LCC and RCC patients, and found that in addition to differences in basic information such as patient age and gender, the differentiation degree differed between the RCC and LCC groups. The RCC group had a lower differentiation degree, which could explain the poorer outcome of RCC patients. The patients underwent adequate examined lymph nodes was more in RCC, suggesting that tumor site affects the examined lymph nodes. This is consistent with Michal's research, which indicated examined lymph nodes was less in LCC patients.^10^

As is mentioned above, metastatic lymph node is a risk prognostic factor for colon cancer, and the N staging system has a strong impact on patient prognosis. In previous studies, like N staging, both LNR and LODDS could predict the outcome of colon patients. N staging, LNR, and the LODDS were closely associated with OS in this study, which is in accordance with previous research.^12^ The differences between the three subs of these stage systems were greater in RCC, and the prognosis of LCC was better no matter in which staging system. The 5‐year OS rates were similar between LCC and RCC. These results indicate that the influence of lymph node status to predict prognosis is greater in RCC patients, which confirms that tumor site can affect lymph node status to predict prognosis. RCC patients had a poorer prognosis than LCC patients, which is consistent with previous studies.^20^ RCC was more insensitive to immunotherapy, which may be related to the fact that RCC is constantly in microsatellite stable/weak immune activation, and it also usually occurs DNA somatic copy number alterations.^7^ In this study, RCC patients had a lower proportion of metastatic lymph nodes, which have an environment that suppresses immune responses.^8^ Therefore, the difference in lymph node status between LCC and RCC might be another reason for the differences in sensitivity to small‐molecule inhibitor immunotherapy aimed at the immune escape.^22^

A good staging system should be precise to predict the outcome of patients.^12^ The C‐index and AIC were performed to estimate the performance of different lymph node staging systems to predict survival of colon cancer patients. We found that LODDS outperformed the other systems in the LCC and RCC groups regardless of^19^whether they were analyzed as categorical or continuous variables. LODDS also performed better than other systems, especially in the TNLN < 12 group. This may compensate for the current deficiency of traditional N staging for assessing prognosis when TNLN is insufficient.^23^ Pei et al.^24^ reported that LODDS showed the best accurate ability to predict the survival of CRC patients. Ye et al. reported similar results in esophageal carcinoma, demonstrating that the prognostic efficacy of LODDS is superior to that of the N descriptor and LNR for estimating OS.^25^ We showed that LCC patients were more in LODDS ≥ −0.9 sub, which might be related to the different sensitivity to immunotherapy between LCC and RCC, and this was not observed in N stage and LNR subs. Above all, these indicate that the discriminative power of LODDS is superior to N stage and LNR.

For most malignant tumors, including colon cancer, nomograms have a more accurate value than the TNM staging to predict the outcome of patients.^19^ Martin et al.^26^ established a nomogram including T stage, number of positive lymph nodes, and postoperative chemotherapy to predict prognosis in colon cancer. Another nomogram for prognosis in colon cancer includes predictors of grade, N stage, T stage, colectomy, and carcinogenic antigen levels.^27^ In this study, LODDS was used to assess lymph node status, and other risk factors were added to build the nomograms in LCC and RCC for predicting OS of colon cancer patients. Validation revealed excellent discrimination and calibration for this nomogram. As shown in the nomogram, T stage and lymph node status (LODDS) were strongly associated with OS. We also found that while T stage was the leading risk factor in LCC, LODDS was the main factor in RCC. This suggests that lymph node status is important for predicting prognosis in RCC patients. We showed that the proportion of patients who underwent adequate examination of lymph nodes differed between the LCC and RCC groups, and we established nomograms of different sites for TNLN < 12 and TNLN ≥ 12. As shown in the nomogram, when the number of regional lymph nodes examined reached 12, LODDS was the leading risk factor. This indicates that regardless of LCC or RCC, when patients undergo adequate examination of 12 lymph nodes, the evaluation of lymph node status is of greater prognostic value. In addition, TNLN < 12 might be a predicting factor of poor prognosis, underscoring the importance of adequate examination of lymph nodes.

As we have seen, this is the first study to compare the prognostic impact of lymph node status in LCC and RCC. Inevitably, some limitations do exist in this research. First, our study is a retrospective study, there are must be some unavoidable selection bias. Second, our study was based on the SEER database, which lacked some clinical information such as the location of metastatic lymph nodes, the drug information of chemotherapy, and the exact number of patients not receive chemotherapy or radiotherapy.^28^, ^29^, ^30^ We used a large training set to construct nomograms and verified them in the validation set, but we still need further validation in prospective clinical trials. Nevertheless, our research was the first to find the influence of lymph node status on predicting prognosis is different between left and right colon cancer patients without metastasis, and we also provide important and effective models for predicting the outcomes in colon cancer patients.

### 4.1 Conclusion

In summary, our study demonstrated that different lymph node status and its influence on prognosis are also an important manifestation of the difference between left‐ and right‐sided colon cancer. We constructed nomograms to predict the prognosis of left‐sided and right‐sided colon cancer patients. Validation experiments showed that the nomograms had precise discriminative ability, accuracy, and clinical effectiveness. In the right‐sided nomogram, lymph node status was the most important factor for predicting prognosis. The results of this study suggest that tumor site needs to be considered when lymph node status is used to assess the outcome of patients. In addition, adequate examination of lymph nodes is necessary for the accurate prediction of patient outcome.

## ETHICS STATEMENT

Data (anonymized) were collected from an available public database and no ethical approval was therefore sought for this study.

## CONFLICT OF INTEREST

The authors have no conflicts of interest to declare.

## DISCLOSURES

The authors declare that no financial relationships exist.

## Supporting information

Fig S1

Fig S2

Fig S3

Fig S4

Fig S5

Fig S6

## Data Availability

The data in this study was collected from an open public database and can be accessed through this link: https://seer.cancer.gov/.
