# Supplementary figures and images for: Lymph node status and its impact on the prognosis of left‐sided and right‐sided colon cancer: A SEER population‐based study
Source: Cancer Med. 2021 Oct 26;10(23):8708–19. doi: 10.1002/cam4.4357 (PMC8633222; doi:10.1002/cam4.4357)

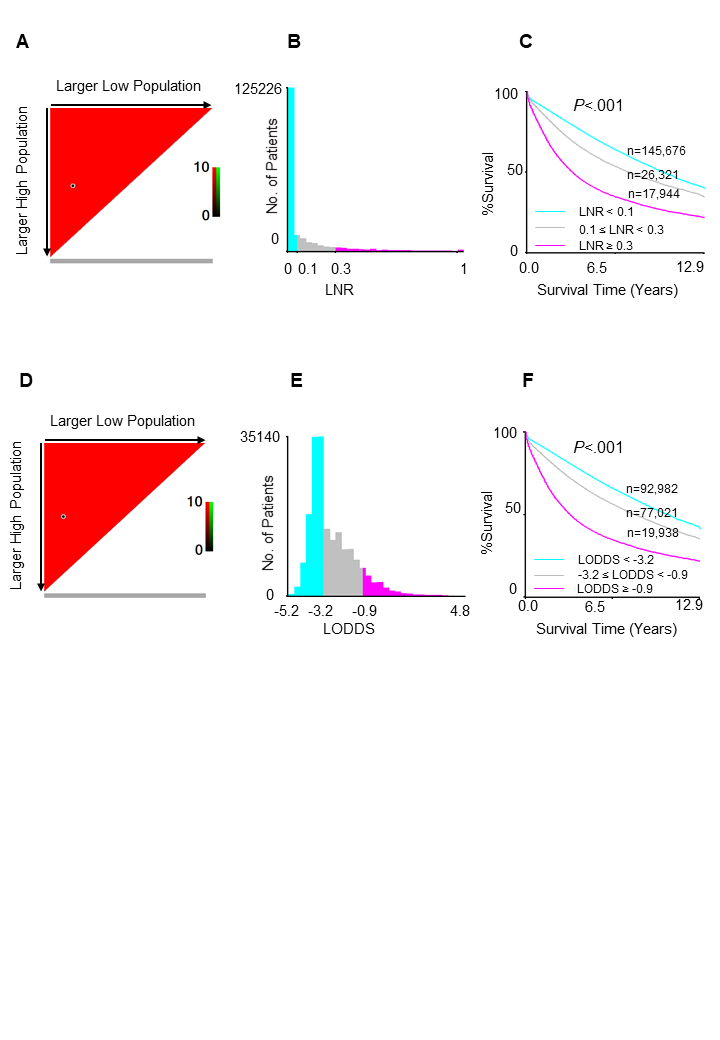

Supplement: Supplementary file 1 — Fig S1 [file CAM4-10-8708-s002.PNG]

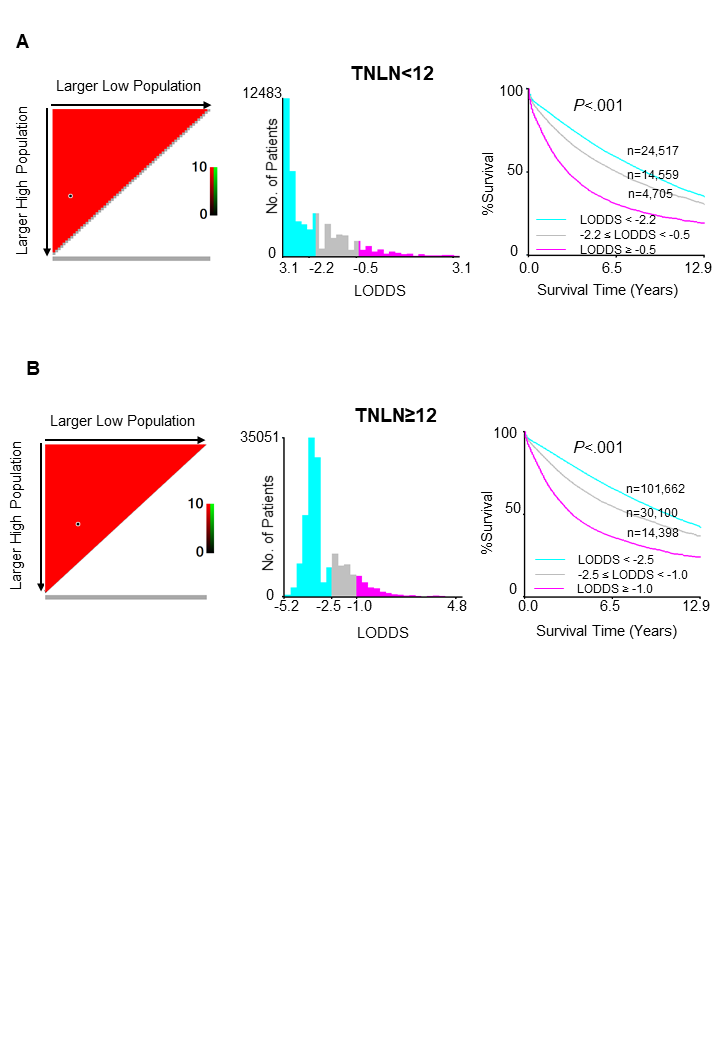

Supplement: Supplementary file 2 — Fig S2 [file CAM4-10-8708-s003.PNG]

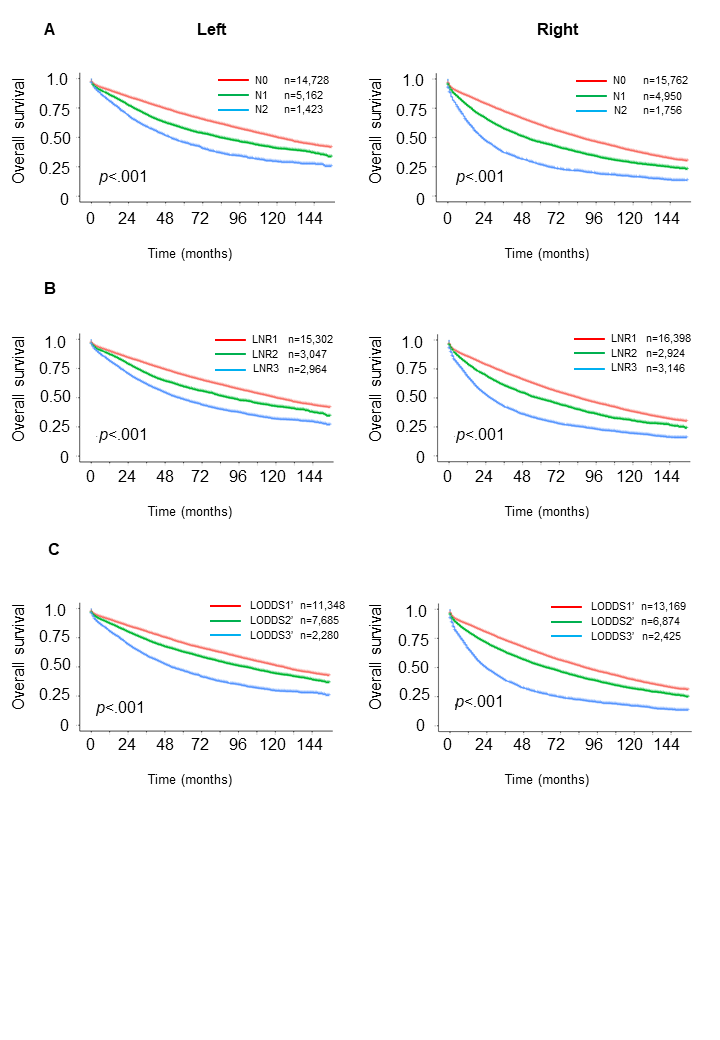

Supplement: Supplementary file 3 — Fig S3 [file CAM4-10-8708-s006.PNG]

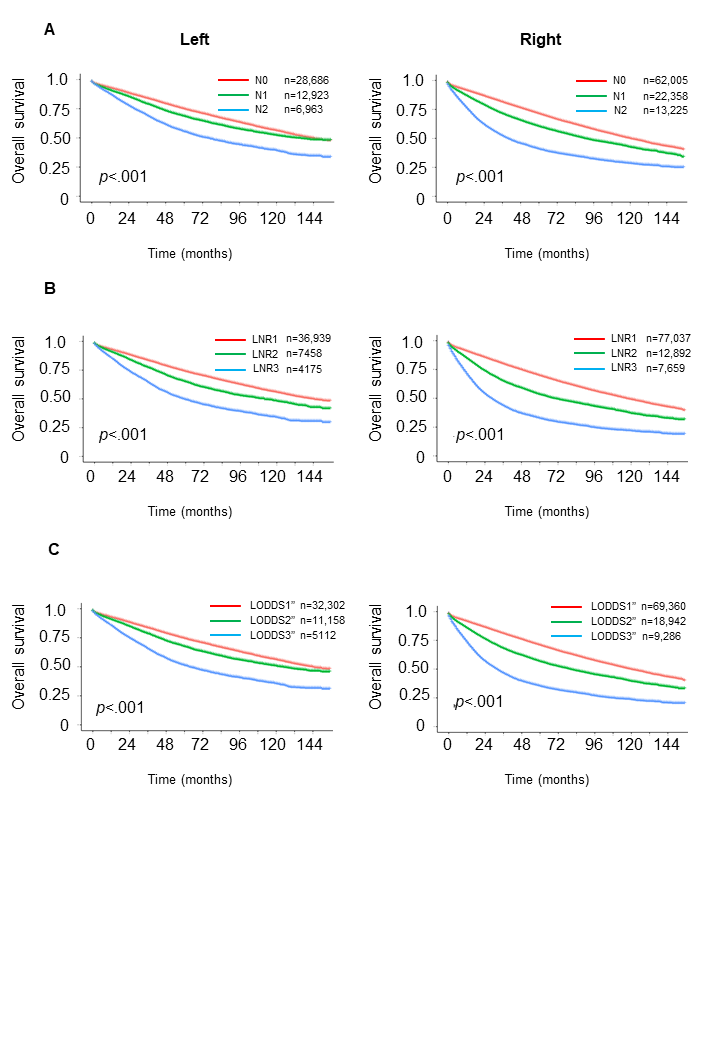

Supplement: Supplementary file 4 — Fig S4 [file CAM4-10-8708-s005.PNG]

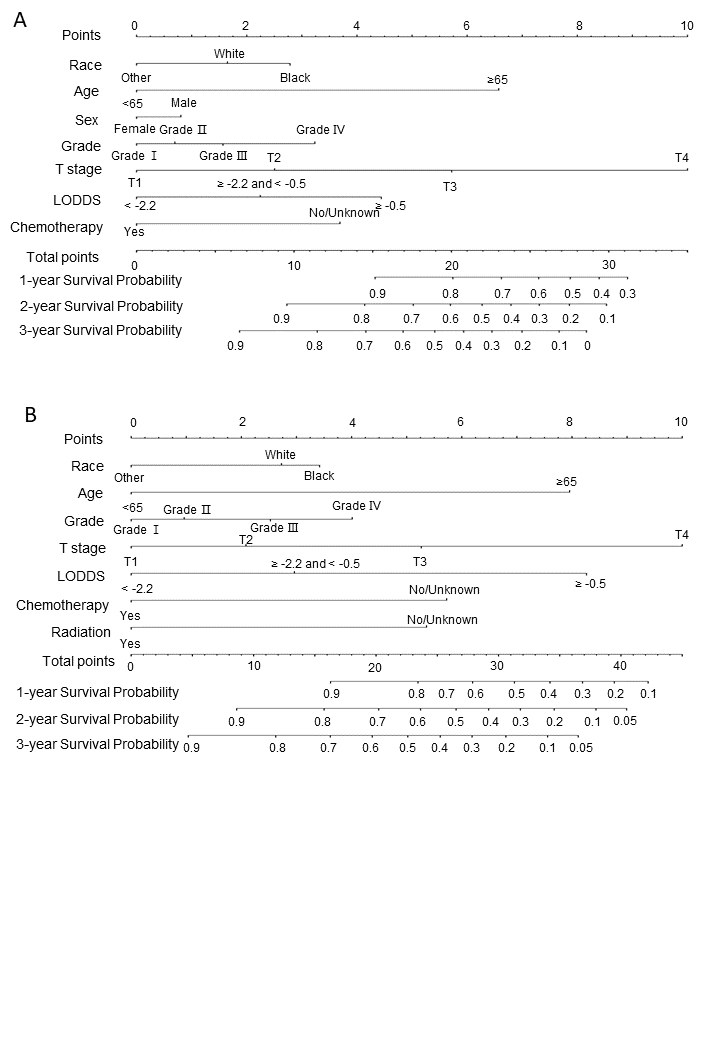

Supplement: Supplementary file 5 — Fig S5 [file CAM4-10-8708-s001.PNG]

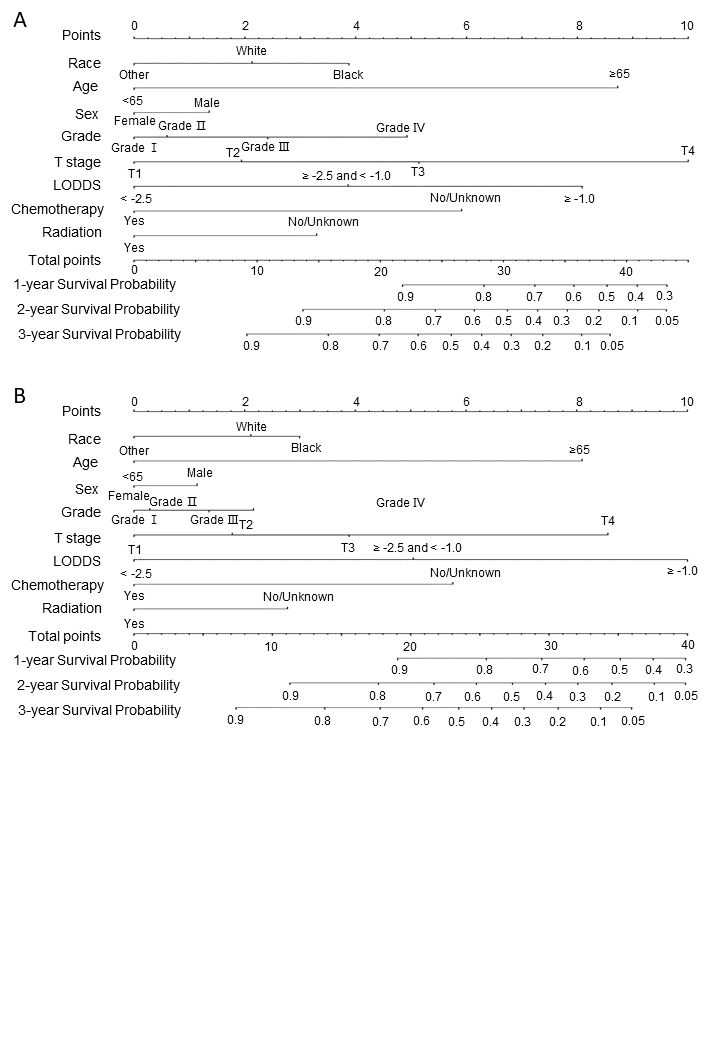

Supplement: Supplementary file 6 — Fig S6 [file CAM4-10-8708-s004.PNG]
